# Peripheral nerve pathology in sickle cell disease mice

**DOI:** 10.1097/PR9.0000000000000765

**Published:** 2019-06-27

**Authors:** Katelyn E. Sadler, Tylor R. Lewis, Tyler B. Waltz, Joseph C. Besharse, Cheryl L. Stucky

**Affiliations:** a Department of Cell Biology, Neurobiology and Anatomy, Medical College of Wisconsin, Milwaukee, WI, USA; b Department of Ophthalmology, Medical College of Wisconsin, Milwaukee, WI, USA

**Keywords:** Sickle cell disease, Myelinopathy, Sciatic nerve

## Abstract

**Introduction:**

Many patients with sickle cell disease (SCD) suffer from chronic pain, which is often described as neuropathic in nature. Although vascular and inflammatory pathology undoubtedly contribute to the SCD pain experience, the nociceptive signals that ultimately drive symptoms are detected and transmitted by peripheral sensory neurons. To date, no systematic histological examination of peripheral nerves has been completed in patients or mouse models of SCD to diagnose disease-related neuropathy.

**Objectives:**

In this brief report, we compared peripheral nerve morphology in tissues obtained from Berkeley transgenic SCD mice and control animals.

**Methods:**

Sciatic nerves were visualized using light and transmission electron microscopy. Myelin basic protein expression was assessed through Western blot. Blood–nerve barrier permeability was measured using Evan's blue plasma extravasation.

**Results:**

Peripheral fibers from SCD mice have thinner myelin sheaths than control mice and widespread myelin instability as evidenced by myelin sheath infolding and unwrapping. Deficits are also observed in nonmyelinating Schwann cell structures; Remak bundles from SCD nerves contain fewer C fibers, some of which are not fully ensheathed by the corresponding Schwann cell. Increased blood–nerve barrier permeability and expression of myelin basic protein are noted in SCD tissue.

**Conclusions:**

These data are the first to characterize Berkeley SCD mice as a naturally occurring model of peripheral neuropathy. Widespread myelin instability is observed in nerves from SCD mice. This pathology may be explained by increased permeability of the blood–nerve barrier and, thus, increased access to circulating demyelinating agents at the level of primary sensory afferents.

## 1. Introduction

Sickle cell disease (SCD) is a hemoglobinopathy associated with many neurological complications including stroke, seizure, and pain.^2^ While pathologically diverse, all these symptoms are ultimately mediated by neurons, the fundamental unit of the nervous system. Despite the collective impact that these neurological symptoms have on patient quality of life, no studies have systematically examined the basic histological properties of neural tissue isolated from either patients with SCD or transgenic SCD mouse models to assess disease-related pathology.

In this report, sciatic nerves from Berkeley transgenic SCD mice (Berk SS) were examined using light and transmission electron microscopy (TEM). The sciatic nerve was chosen for these studies because of its large size and involvement in peripheral somatosensation. Dysregulated sensory systems lead to intense pain which is the leading cause of emergency department visits for patients with SCD.^28^ In both mouse and human, the sciatic nerve contains the thickly myelinated axons of motor efferents and the thinly myelinated and unmyelinated axons of sensory afferents, many of which are nociceptors.^21^ Here, we report skewed myelinated axon diameter distributions and decreased Remak bundle C-fiber density in Berk SS nerves. We also report decreased myelin sheath thickness and aberrant myelin sheath structures in nerves isolated from Berk SS mice. These changes are accompanied by an increase in myelin basic protein (MBP) expression and blood–nerve barrier permeability. These data are the first demonstration of myelin and axonal changes in SCD neural tissues, a phenotype that may contribute to many common SCD complications.

## 2. Methods

### 2.1. Mouse model and tissue collection

Berkeley transgenic mice homozygous for the sickle β globin gene were compared with control B6; 129 mice.^19^ Sciatic nerves (10 mm length isolated ∼5 mm distal to hip) were obtained from male and female mice aged 8 to 12 weeks. All protocols were in accordance with National Institutes of Health guidelines and approved by the Institutional Animal Care and Use Committee at the Medical College of Wisconsin.

### 2.2. Light and transmission electron microscopy

Freshly dissected sciatic nerves were prepared as previously described.^14^ For light microscopy, semithin sections (0.5 µm) stained with toluidine blue were imaged on a Nikon Eclipse TE300 microscope. For TEM, thin sections (70 nm) were stained with uranyl acetate and lead citrate, then imaged on a Hitachi H-600 transmission electron microscope. Image analysis was completed using ImageJ software.

### 2.3. Myelin basic protein Western blot

Sciatic nerves were snap frozen on dry ice, pooled for individual animals then homogenized in lysis buffer (50 mM Tris base pH 8, 0.5% NP-40, 10% glycerol, 0.1 mM EDTA, 250 mM NaCl, 1X Halt protease inhibitor cocktail). Total protein content was analyzed using a BCA Protein Assay Kit (Pierce; ThermoFisher, Waltham, MA); 20 µg of protein was separated on 4% to 12% Bis–Tris gradient gels and transferred to nitrocellulose membranes. Membranes were blocked in Odyssey blocking buffer for 1 hour and incubated with mouse anti-MBP (abcam #ab62631, 1:1000; Abcam, Cambridge, United Kingdom) and rabbit anti-β-tubulin (abcam #ab6046, 1:10,000) primary antibodies for 1 hour. Blots were washed with 0.1% Tween-20 in PBS and incubated with donkey anti-rabbit 800 (LI-COR #926-32213, 1:15,000) and donkey anti-mouse 680 (LI-COR #926-68072, 1:15,000) secondary antibodies for 1 hour. Blots were scanned on an Odyssey infrared imaging system, and band densitometry was assessed using Image Studio Lite software. For each sample, MBP (23 and 18 kDa bands) was normalized to β-tubulin; SCD samples were then normalized to control average.

### 2.4. Evan's blue extravasation

Evan's blue (50 mg/mL stock, 80 mg/g body weight) was injected into the tail vein. Fifteen minutes after injection, mice were sacrificed and both sciatic nerves were harvested, weighed, and snap frozen. Nerves were incubated in 50% TCA (1 mg tissue: 1 μL solution) for 5 minutes then homogenized. Supernatant absorbance was measured at 620 nm, and Evan's blue concentration was interpolated from a standard curve.

### 2.5. Data analysis

All data were analyzed by an experimenter blinded to genotype. In the interest of data transparency, individual data points were presented in addition to group mean ± SEM. Two group data sets were analyzed through unpaired *t* test. Axon diameter distributions were compared through χ^2^ analysis followed by corrected Fisher's exact tests. G ratios were analyzed through linear regression. Data were analyzed using GraphPad Prism 6; results were considered statistically significant when *P* < 0.05.

## 3. Results

**Figure 1.**
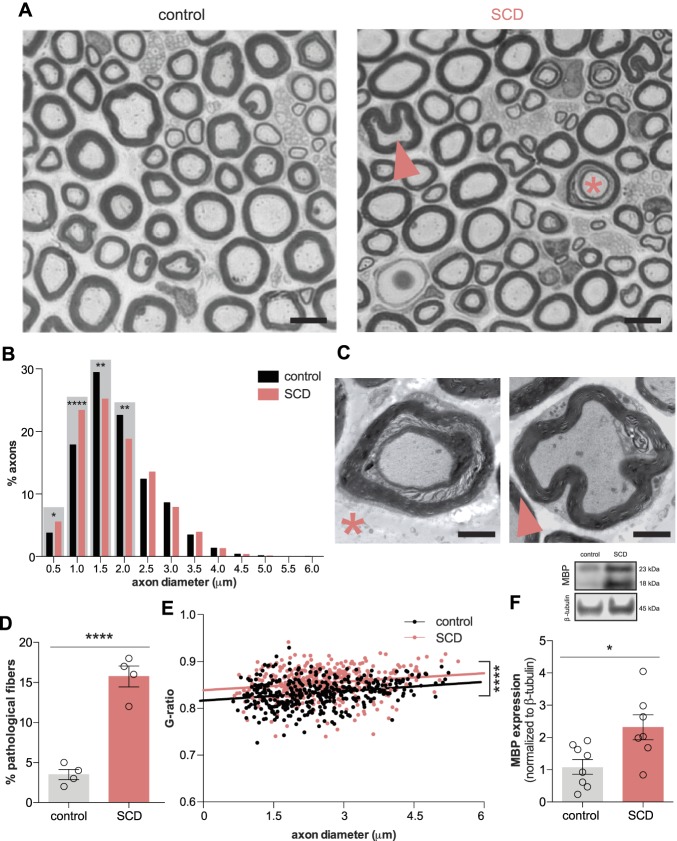
Myelinated fiber pathology in SCD mice. (A) Pathological fibers (Δfiber with myelin sheath infolding; *fiber with unraveling myelin sheath) are observed in cross-sections of SCD sciatic nerve (scale bar = 2 μm). (B) Distribution of myelinated axon diameters in control and SCD mice (χ^2^ *P* < 0.0001; Fisher's exact test *P < 0.05, **P < 0.01, ****P < 0.0001; n = 4 biological replicates; n = 100 fibers/biological replicate). (C) Representative TEM images of pathological fibers (*unraveling myelin sheaths; Δmyelin sheath infolding; scale bar = 2 μm). (D) Increased prevalence of pathological fibers is observed in sciatic nerves from SCD mice (unpaired *t* test ****P < 0.0001) (E) Increased G ratios (ie, thinner myelin sheaths) are observed in SCD fibers of all diameters (linear regression ****P < 0.0001). (F) When normalized to β-tubulin, Western blots reveal increased MBP level in SCD sciatic nerves (unpaired *t* test *P < 0.05; n = 7–8). MBP, myelin basic protein; TEM, transmission electron microscopy; SCD, sickle cell disease.

Gross changes in SCD-myelinated nerve histology were assessed using light microscopy (Fig. 1A). Berk SS nerves contained a greater proportion of the smallest diameter myelinated fibers (0.5–1.0 μm) and a smaller proportion of 1.5 to 2.0 μm diameter fibers than control nerves; no differences were noted in large diameter fiber abundance (Fig. 1B). Increased numbers of pathological myelin patterns were observed in SCD nerves (Figure 1A, C, D). Myelin sheath infolding and ring separation were the most commonly observed patterns. Thinner myelin sheaths (indicated by increased G ratios of axon diameter/myelinated fiber diameter) were observed in SCD nerves (Fig. 1E). Western blotting revealed increased MBP expression in SCD nerves (Fig. 1F), suggesting that resident Schwann cells are actively myelinating peripheral fibers^10^ in SCD so as to replace unwrapping myelin sheaths.

**Figure 2.**
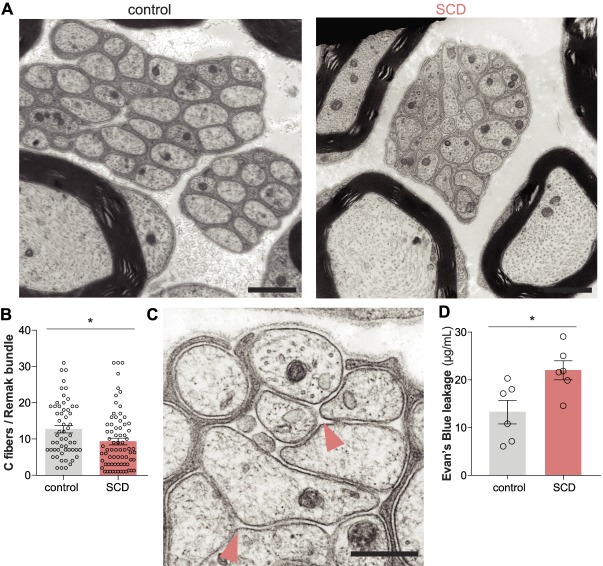
Unmyelinated fiber pathology in SCD mice. (A) Unmyelinated C-fiber pathology was examined through TEM in cross-sections of control and SCD sciatic nerves (scale bars = 1 μm). (B) Fewer C fibers were contained within the Remak bundles of SCD nerves (unpaired *t* test *P < 0.05; n = 4 biological replicates). (C) Representative image of SCD Remak bundle in which nonmyelinating Schwann cell has failed to complete ensheath C fibers (Δpotential points of direct contact between fibers; scale bar = 500 nm). (D) Increased permeability of the blood–nerve barrier is observed in SCD mice as measured by Evan's blue plasma extravasation (unpaired *t* test *P < 0.05; n = 6). SCD, sickle cell disease; TEM, transmission electron microscopy.

Gross changes in SCD C fiber histology were assessed using TEM (Fig. 2A). In SCD nerves, fewer C fibers were packaged per Remak bundle (Fig. 2B). Nonmyelinating Schwann cells also occasionally failed to completely ensheath C fiber axons in SCD nerves (Fig. 2C). Both the nonmyelinated and myelinated peripheral fiber pathology in SCD tissue may have resulted from the increased permeability of the blood–nerve barrier observed through Evan's blue plasma extravasation (Fig. 2D).

## 4. Discussion

Many patients with SCD describe their pain symptoms using neuropathic qualifiers.^3^ Similarly, transgenic mouse models of SCD display neuropathic pain-like behaviors that are similar to those observed in patients with SCD.^8,22^ Here, we present the first histological evidence that characterize Berkeley SCD mice as a naturally occurring model of polyneuropathy. To date, nerve biopsies from patients with SCD have only been presented in 1 case report; qualitative analysis revealed thinner than normal myelin sheaths, segmental demyelination, and myelin ovoids in the sural nerves of patients with SCD, suggesting a similar widespread myelin instability phenotype in both patients and mice with SCD. However, individuals examined in the case report were also receiving sodium cyanate therapy, a drug with known myelinotoxic effects,^20^ and therefore, generalizations about the prevalence of this phenotype in the broader patient population should not be made. Notably, while not apparent in SCD mice, decreased conduction velocity, a possible electrophysiological outcome of reduced myelin thickness, has been reported in SCD patient nerves.^1,17^

In addition to decreased thickness, the myelin sheath of many SCD mouse fibers appeared unstable; myelin sheath infolding and ring separation, patterns commonly observed in nerve biopsies from patients and mouse models with polyneuropathy,^4,13,18,27^ were frequently observed in SCD tissue. Increased MBP expression was noted in SCD nerves suggesting ongoing myelination; validation of these processes should be addressed with additional myelin-associated protein expression profiling. Regardless, when considered with the peripheral nerve sprouting and irregular epidermal innervation qualitatively observed in these mice by other groups,^11^ these data strongly suggest that the chronic pain-like behaviors exhibited by SCD mice are generated, in part, through peripheral neuropathic mechanisms. These data are further supported by electrophysiological recordings performed in SCD mice. Functionally identified Aβ, Aδ, and C fibers from SCD mice exhibit decreased stimulation thresholds and increased firing upon receptive field stimulation.^7,9,23^ C fibers from SCD mice also exhibit high levels of spontaneous activity.^23^ Here, we report that in SCD tissue, nonmyelinating Schwann cells sometimes fail to completely ensheath C fibers contained within a given Remak bundle. This absence of complete fiber insulation may lead to cross‐excitability or ephaptic firing between axons,^6,15^ thereby resulting in the increased C-fiber “spontaneous” activity and increased pain-like behaviors observed in SCD mice.^23^

In patients with SCD, additional evidence for myelinopathy comes from hyperintensities of cerebral white matter observed through magnetic resonance imaging.^12^ These imaging abnormalities, which are correlates of demyelination and axonal degradation, may arise from silent cerebral infarcts that are common in SCD,^2^ or be subclinical indicators of widespread neuronal instability. Neuronal destabilization in this disease may result from autoimmune mechanisms such as those observed in multiple sclerosis,^16^ another chronic proinflammatory disease,^5^ or from circulating factors leaking through the highly permeable blood–nervous system barrier demonstrated here in SCD mice. Such circulating factors include lysophosphatidylcholine, a membrane lipid and known demyelinating agent^25^ that is increased in serum from patients and mice with SCD,^26^ and free hemoglobin, a chemical released from sickled red blood cells that has neurotoxic effects.^24^ Although histological examination of patient tissue is needed to validate these preclinical finding, data like these will aid in the development of novel therapeutics that target the root cause of many SCD phenotypes, rather than simply alleviating disease symptoms such as pain.

## Disclosures

The authors have no conflict of interest to declare.

This study was supported by research funding from the National Institutes of Health to K.E. Sadler (grant F32NS106789) and C.L. Stucky (grants NS040538 and NS070711).
